# Acute general hospital admissions in people with serious mental illness

**DOI:** 10.1017/S0033291718000284

**Published:** 2018-02-28

**Authors:** Nishamali Jayatilleke, Richard D. Hayes, Chin-Kuo Chang, Robert Stewart

**Affiliations:** 1 King's College London (Institute of Psychiatry, Psychology, and Neuroscience), UK; 2 Biomedical Research Centre Nucleus, South London and Maudsley NHS Foundation Trust, London, UK; 3 Department of Health and Welfare, University of Taipei, Taipei City, Taiwan

**Keywords:** Bipolar disorder, health service, physical illness, schizoaffective disorder, schizophrenia

## Abstract

**Background:**

Serious mental illness (SMI, including schizophrenia, schizoaffective disorder, and bipolar disorder) is associated with worse general health. However, admissions to general hospitals have received little investigation. We sought to delineate frequencies of and causes for non-psychiatric hospital admissions in SMI and compare with the general population in the same area.

**Methods:**

Records of 18 380 individuals with SMI aged ⩾20 years in southeast London were linked to hospitalisation data. Age- and gender-standardised admission ratios (SARs) were calculated by primary discharge diagnoses in the 10th edition of the World Health Organization International Classification of Diseases (ICD-10) codes, referencing geographic catchment data.

**Results:**

Commonest discharge diagnosis categories in the SMI cohort were urinary conditions, digestive conditions, unclassified symptoms, neoplasms, and respiratory conditions. SARs were raised for most major categories, except neoplasms for a significantly lower risk. Hospitalisation risks were specifically higher for poisoning and external causes, injury, endocrine/metabolic conditions, haematological, neurological, dermatological, infectious and non-specific (‘Z-code’) causes. The five commonest specific ICD-10 diagnoses at discharge were ‘chronic renal failure’ (N18), a non-specific code (Z04), ‘dental caries’ (K02), ‘other disorders of the urinary system’ (N39), and ‘pain in throat and chest’ (R07), all of which were higher than expected (SARs ranging 1.57–6.66).

**Conclusion:**

A range of reasons for non-psychiatric hospitalisation in SMI is apparent, with self-harm, self-neglect and/or reduced healthcare access, and medically unexplained symptoms as potential underlying explanations.

## Introduction

There is a growing awareness of the adverse physical health experienced by people living with serious mental illness (SMI, which includes schizophrenia, schizoaffective disorder, and bipolar disorder). Specifically, people with SMI, experience more medical comorbidities than the general population (Leucht *et al.* 2007; De Hert *et al.* 2011), resulting in substantially higher mortality and shortened life expectancy (Chang *et al*. 2011; Lawrence *et al*. 2013). Nearly half of people with any mental disorder have comorbid medical problems, and a further 35% have undiagnosed medical conditions, implying that only a small proportion are free of comorbidities (Miller *et al.* 2006; Zolnierek *et al.* 2009). As reviewed previously (Miller *et al.* 2006), this is particularly an issue for patients with SMI, with the risk for diabetes being doubled for patients with schizophrenia and three times higher for patients with bipolar disorder. Similarly, cardiovascular disease is increased 2–3 folds in prevalence, with a particular impact on younger adults. Individuals with SMI are also at increased risk of pneumonia and need for mechanical ventilation, and have a raised risk of emphysema, even after controlling smoking (Miller *et al*. 2006). Studies examining specific medical comorbidities among patients with SMI have reported nearly doubled odds of cardiovascular, endocrine, and respiratory conditions (Bahorik *et al*. 2017). Compared with controls, individuals with bipolar disorder were significantly less likely to have no recorded physical conditions (OR 0.59, 95% CI 0.54–0.63) and significantly more likely to have one physical condition (OR 1.27, 95% CI 1.16–1.39), two physical conditions (OR 1.45, 95% CI 1.30–1.62) and three or more physical conditions (OR 1.44, 95% CI 1.30–1.64) (Smith *et al*. 2013*a*, *b*). Compared with controls, people with schizophrenia were significantly more likely to have one physical-health comorbidity (OR 1.21, 95% CI 1.16–1.27), two physical-health comorbidities (OR 1.37, 95% CI 1.29–1.44), and three or more physical-health comorbidities (OR 1.19, 95% CI 1.12–1.27) (Smith *et al*. 2013*a*, *b*). Some of these conditions themselves may impact the mental condition by exacerbating signs and symptoms, response to psychotropic drugs, life expectancy, and access to healthcare services. The number of comorbid health conditions was associated with higher odds of using speciality mental health service, while not associated with utilisation of services provided by general health care providers (Lee, 2016).

In the UK, most people with SMI live in community accommodation and receive public universal healthcare in the same centres used by the general population (Health and Social Care Information Centre, 2013). More investigation is warranted given people with SMI are known to be at high risk of a number of physical illnesses which cover multiple disease categories and body systems (Jones *et al*. 2004) and there is no consensus on how to treat or prevent physical disease in this group (Mitchell *et al*. 2012).

### Aims of the study

Levels of and reasons for the use of acute care (i.e. general hospital/secondary physical healthcare) services have not been adequately characterised for people with SMI, despite the importance of this information for primary care and other community preventative services, as well as acute care providers. Using a large linked database of mental health and acute care records, we, therefore, sought to describe and investigate the commonly recorded reasons for acute care hospitalisation in people with SMI.

## Material and methods

### Setting

The South London and Maudsley NHS Foundation Trust (SLaM) is one of Europe's largest providers of secondary mental healthcare, serving a population of approximately 1.36 million residents in four London boroughs (Lambeth, Southwark, Lewisham and Croydon) as a near-monopoly source of comprehensive secondary mental healthcare including inpatient, community, general hospital liaison, and forensic services. From 2006 onwards, electronic clinical records have been used comprehensively across all SLaM services, and in 2008, the Clinical Record Interactive Search (CRIS) system supported by SLaM's NIHR Biomedical Research Centre for Mental Health was developed to enable researchers to search and retrieve anonymised electronic health records efficiently. CRIS currently provides anonymised in-depth mental healthcare information derived for over 287 000 service users. The protocol for this data resource has been previously described in detail (Stewart *et al*. 2009; Fernandes *et al.* 2013; Perera *et al*. 2016) and CRIS is approved as a dataset for secondary analysis (Oxfordshire Research Ethics Committee C, reference 08/H0606/71+5).

### Sample and outcomes

Cohort members were defined on the basis of a diagnosis of schizophrenia (ICD-10 code: F20), schizoaffective disorder (F25) or bipolar affective disorder (F31) recorded by SLaM on or before the 31 December 2006 and on the basis of at least one contact with SLaM during 2007 or 2008. Diagnoses recorded in CRIS are based on the 10th edition of the World Health Organization International Classification of Diseases (ICD-10) and were extracted both from those recorded in structured drop-down menus within the source electronic health record or extracted from free text fields using bespoke software (Perera *et al*. 2016). The grouping of SMI has been maintained to remain in line with other recent publications (Chang *et al.* 2011; Hayes *et al*. 2012). Non-psychiatric hospitalisations were investigated over an observation period from 1 January 2009 to 31 December 2010 inclusive, using a data linkage between CRIS and Hospital Episode Statistics (HES) (Perera *et al*. 2016).

In the UK, HES data are compiled for all healthcare providers in England (both acute and mental health services), including statistical abstracts of records of all inpatient episodes, as well as outpatient and emergency care. A dataset has been compiled which contains all HES data for SLaM's catchment with a linkage to CRIS data, described in detail elsewhere (Perera *et al*. 2016), and allowing standardised morbidity statistics to be calculated for people with mental disorders on the CRIS database in reference to the catchment general population. Individuals who were under the age of 20 at the start of the observation period or when they were first diagnosed as SMI during the period were excluded from the analysis, and admissions to any mental health inpatient unit were excluded as outcomes.

Three-character ICD-10 codes for listed discharge diagnoses were extracted for each hospitalisation. A hospitalisation was defined as having at least one HES episode and contiguous episodes (i.e. where start and end dates were on the same day) were combined where necessary into single hospitalisations. Only the final primary discharge diagnosis of each admission (i.e. that applying to the last episode of that admission, where multiple episodes were involved) was used. Age in the analysis was defined at the date of admission for physical illness where this occurred or at the mid-point of each 1-year observation period for those not admitted in a specific year.

### Diagnosis groupings and data management

The grouping of diagnoses was carried out in line roughly with the chapters of the International Statistical Classification of Diseases and Related Health Problems, 10th revision (ICD-10), either by type of conditions (for example, cancers grouped together) or by organ affected, such as conditions of the eye (World Health Organization, 2010).

For this study, data were obtained for the SMI cohort and comparator population using the CRIS-HES linkage. The age and gender profile for the SMI cohort were obtained on 1 January 2010 as the mid-point of the observation period for hospitalisations, and the age and gender profile of the comparator population were obtained from the 2011 UK census as this was the nearest to the observation period. Discharge diagnoses (one per hospitalisation; primary diagnosis if multiple) were obtained for the SMI cohort in 2009 and 2010. Age and gender were accounted for through indirect standardisation and the generation of standardised admission ratios (SARs). We carried out an additional stratification by affective (bipolar/schizoaffective disorders) and non-affective (schizophrenia) SMI diagnosis.

### Statistical analysis

Age- and sex-SARs were calculated for the 2 years of the observation period (2009–2010) for the cohort with SMI by Stata12. Using the observed number of specific admissions as the numerator, the denominator was the expected number of admissions in the same period of time, estimated using the age- and gender-specific admissions rates in 2009 and 2010 for SLaM's catchment area. SARs were calculated first for diagnoses grouped at the highest level (letter) ICD-10 codes: these relate predominantly to the body system affected or type of disorder, and were grouped/split as follows: (i) splitting urinary (N00-N39) from other (N40+) genitourinary conditions; (ii) combining neoplasms (all C codes and D00-D48) and separating these from blood disorders (D50+); (iii) combining injuries and external causes (all S codes and T00-T14) and separating these from poisoning (T15+); (iv) combining infections (all A and B codes). Analyses were finally carried for more specific three-character diagnostic codes, describing those accounting for at least 1% of all hospitalisations in the SMI cohort. We then excluded repeat admissions for the same ICD-10 three-character code for the disease groups and re-calculated ratios as sensitivity analyses. Adjustments for multiple comparisons were not carried out as the estimates only sought to provide descriptive information.

## Results

The SMI cohort at the South London & Maudsley NHS Foundation Trust (SLaM hospital) consisted of 10 049 males and 8331 females at mid-point of the follow-up period on 1 January 2010. There were 8622 admissions among individuals with a diagnosis of schizophrenia, schizoaffective disorder, or bipolar disorder who were observed from 1 January 2009 to 31 December 2010. In the comparison population (residents of the SLaM catchment areas), the total number of admissions was 501 158 (among the 1.36 million population in 2011 Census data). Table 1 displays numbers of hospitalisations by general chapters of ICD-10 codes, and age- and sex-SARs in the cohort. The top five primary causes for hospitalisation were urinary conditions (ICD-10 codes: N00-N39), digestive conditions (K00-K93), unclassified symptoms (R00-R99), neoplasms (C00-D48), and respiratory conditions (J00- J99). SARs were raised for all of these apart from that for neoplasms where admissions were remarkably lower in the cohort. People with SMI also had higher risks of admission than the general population for poisoning and external causes, injury, mental and behavioural disorders, endocrine/metabolic conditions, blood disorders, nervous disorders, skin disorders and infections, as well as ‘Z-codes’ (another non-specific cause). Only neoplasms, musculoskeletal disorders, pregnancy-related disorders, and eye disorders showed significantly lower SARs.

**Table 1.** Age- and gender-standardised admission ratios (SARs) for hospitalisations in 2009–2010 in people with serious mental illness (*N* of all admissions = 8622), compared with the source population

Primary cause of admission (ICD-10 codes)	Number of hospitalisations in the SMI cohort	SAR (95% CI)
Genitourinary system: urinary conditions (N00-N39)	1886	1.60 (1.53–1.67)
Digestive system (K00-K93)	974	1.51 (1.41–1.60)
Symptoms, signs, and findings, not elsewhere classified (R00-R99)	898	1.91 (1.79–2.04)
Neoplasms (C00-D48)	632	0.66 (0.61–0.71)
Respiratory system (J00-J99)	511	2.57 (2.35–2.80)
Factors influencing health status and contact with health services (Z00-Z99)	453	1.72 (1.57–1.89)
Circulatory system (I00-I99)	444	1.08 (0.98–1.18)
Poisoning and other external causes (T15-T98)	368	5.03 (4.53–5.57)
Injury (S00-T14)	352	2.52 (2.27–2.80)
Mental and behavioural disorders (F00-F99)	299	30.3 (27.0–34.0)
Musculoskeletal system (M00-M99)	280	0.77 (0.69–0.87)
Endocrine and metabolic diseases (E00-E90)	277	3.07 (2.72–3.45)
Pregnancy related (O00-O99)	256	0.84 (0.74–0.95)
Blood disorders (D50-D89)	236	2.13 (1.87–2.42)
Nervous system (G00-G99)	176	1.94 (1.66–2.25)
Genitourinary system: pelvis, genitals, and breasts (N40-N99)	165	0.98 (0.83–1.14)
Eye conditions (H00-H59)	162	0.61 (0.52–0.72)
Skin conditions (L00-L99)	159	1.54 (1.31–1.80)
Infectious diseases (A00-B99)	78	2.06 (1.63–2.58)
Ear conditions (H60-H95)	10	0.77 (0.37–1.42)
Congenital abnormalities (Q00-Q99)	6	0.67 (0.25–1.46)

Table 2 displays re-calculated age- and sex-SARs for the SMI cohort, excluding repeat admissions (defined as primary discharge diagnoses falling within the same grouped codes in the 2-year follow-up period). Here, the five most common reasons for admission, using general chapters of ICD-10 codes, were digestive, unclassified symptoms, respiratory conditions, ‘Z-codes’ and circulatory disorders. While the rankings were different from Table 1 in terms of the frequency of hospitalisations for SMI, the direction and significance of the SARs remained. Ratios of all admissions (Table 1) to those excluding repeat admissions (Table 2) in people with SMI were highest for urinary conditions, neoplasms, and blood disorders, indicating high numbers of repeat hospitalisations in those diagnostic groups.

**Table 2.** Age- and gender-standardised admission ratios (SARs) for people with serious mental illness excluding repeat hospitalisations (*N* of subjects with admissions = 5377)

Primary cause of admission (ICD-10 codes)	Number of SMI subjects admitted (ratio of all admissions to unique admissions)	SARs (95% CI)
Digestive system (K00-K93)	780 (1.25)	1.37 (1.28–1.47)
Symptoms, signs, and findings, not elsewhere classified (R00-R99)	752 (1.19)	1.78 (1.65–1.91)
Respiratory system (J00-J99)	409 (1.25)	2.55 (2.30–2.80)
Factors influencing health status and contact with health services (Z00-Z99)	378 (1.20)	1.73 (1.55–1.91)
Circulatory system (I00-I99)	347 (1.28)	1.06 (0.95–1.18)
Injury (S00-T14)	331 (1.06)	2.52 (2.26–2.81)
Mental and behavioural conditions (F00-F99)	299 (1.00)	30.3 (27.0–34.0)
Poisoning and other external causes (T15-T98)	275 (1.34)	4.59 (4.07–5.17)
Genitourinary system: urinary conditions (N00-N39)	257 (7.34)	1.67 (1.47–1.89)
Musculoskeletal system (M00-M99)	237 (1.18)	0.80 (0.70–0.91)
Pregnancy related (O00-O99)	217 (1.18)	0.80 (0.70–0.92)
Endocrine and metabolic diseases (E00-E90)	205 (1.35)	3.49 (3.02–4.00)
Neoplasms (C00-D48)	202 (3.13)	0.70 (0.60–0.80)
Genitourinary system: pelvis, genitals, and breasts (N40-N99)	140 (1.18)	0.92 (0.78–1.09)
Skin conditions (L00-L99)	138 (1.15)	1.53 (1.29–1.81)
Nervous system (G00-G99)	126 (1.40)	1.82 (1.51–2.16)
Eye conditions (H00-H59)	120 (1.35)	0.62 (0.52–0.74)
Blood disorders (D50-D89)	83 (2.84)	1.41 (1.12–1.74)
Infectious diseases (A00-B99)	65 (1.20)	1.91 (1.48–2.44)
Ear conditions (H60-H95)	10 (1.00)	0.82 (0.39–1.50)
Congenital abnormalities (Q00-Q99)	6 (1.00)	0.78 (0.29–1.70)

Table 3 displays the three-character ICD-10 diagnoses which accounted for at least 1% of all primary discharge diagnoses, and indicates that a few conditions accounted for multiple hospitalisation instances in the SMI cohort. The most common was a chronic renal failure (N18) accounting for 18.5% of all admissions, but this was substantially less common as a cause of any admission (i.e. when repeat admissions were excluded), as the 1592 hospitalisations occurred in only 22 individuals. Dental caries, other disorders of the urinary system, pneumonia, sickle cell disorders, type 2 diabetes mellitus, and chronic obstructive pulmonary disease were other common specific disorder diagnoses with raised SARs, in addition to non-specific diagnoses such as throat/chest pain, abdominal/pelvic pain, the Z04 code, and alcohol-related presentation. Apart from alcohol-related presentations (F10), all SARs remained statistically significant when repeat admissions were excluded. ‘Other cataract’ was the only one of these hospitalisation diagnoses which was less common in SMI than in the source population, and breast cancer the only diagnosis showing no statistically significant difference in admission rates. Considering ratios of all admissions to unique admissions in SMI (i.e. column 3 divided by column 5 in Table 3), the highest values were found for chronic renal failure (72.4), sickle cell disorders (7.6), breast cancer (5.7), and disorders related to alcohol use (2.0), with the remainder being below 1.5.

**Table 3.** Age- and gender-standardised admission ratios (SARs) for three-character ICD code diagnoses contributing to at least 1% of all hospitalisations in 2009–2010 among patients with SMI in southeast London

ICD-10 code	Denoting clinical syndrome	All hospitalisations		Excluding repeat hospitalisations	
		Number (% of total)	Standardised admission ratio (95% CI)	Number	SAR
N18	Chronic renal failure	1592 (18.5)	1.60 (1.53–1.68)	22	1.69 (1.06–2.56)
Z04	Examination and observation for other reasons	264 (3.1)	6.66 (5.88–7.51)	211	5.60 (4.87–6.41)
K02	Dental caries	222 (2.6)	2.55 (2.22–2.91)	159	2.24 (1.91–2.62)
N39	Other disorders of urinary system	173 (2.0)	2.65 (2.27–3.07)	137	2.55 (2.14–3.01)
R07	Pain in throat and chest	149 (1.7)	1.57 (1.33–1.85)	110	1.37 (1.12–1.65)
J18	Pneumonia, organism unspecified	144 (1.7)	3.33 (2.81–3.92)	126	3.26 (2.72–3.88)
C50	Malignant neoplasm of breast	120 (1.4)	0.87 (0.72–1.04)	21	0.95 (0.59–1.45)
R10	Abdominal and pelvic pain	115 (1.3)	1.56 (1.29–1.87)	84	1.27 (1.01–1.58)
D57	Sickle-cell disorders	114 (1.3)	9.04 (7.45–10.9)	15	4.83 (2.70–7.97)
E11	Type 2 diabetes mellitus	101 (1.2)	4.46 (3.63–5.42)	73	4.28 (3.35–5..38)
F10	Mental and behavioural disorders due to use of alcohol	91 (1.1)	16.5 (13.3–20.2)	45	9.80 (0.15–13.1)
J44	Other chronic obstructive pulmonary disease	88 (1.0)	1.85 (1.48–2.28)	62	2.18 (1.67–2.79)
H26	Other cataract	87 (1.0)	0.49 (0.39–0.60)	63	0.50 (0.39–0.64)

Stratifications of analyses by affective/non-affective SMI diagnosis are provided in online Supplementary Tables S1–S3. In general, most of the SARs were similar between groups. Of differences observed, miscellaneous diagnoses (R- and Z-codes) were more strongly associated with non-affective disorders in analyses excluding repeat admissions (see online Supplementary Table S2), as were a number of the specific diagnoses – notably chronic renal failure, dental caries, urinary system disorders (N39), type 2 diabetes mellitus, as well as the non-specific codes of throat/chest pain (R07) and Z04. Only alcohol-related disorder (F10) was more common as a diagnosis in affective compared with non-affective disorders.

## Discussion

In this study, we sought to draw a profile of the recorded reasons for non-psychiatric hospital admissions in people with SMI. In terms of overall numbers of hospital admissions in SMI, the leading diagnostic groups represented urinary, digestive, neoplastic, respiratory, and circulatory conditions. For all of these groups of conditions apart from circulatory disease, admission rates were significantly different to those from the general population, with SARs higher than population levels for urinary, digestive and respiratory conditions and lower for neoplasms. Hospitalisations with non-specific diagnostic categories (ICD-10 R and Z codes) were also common and higher than in the general population as were those due to injuries, poisonings, and other external causes, and those categorised as due to mental disorder. All of these observations held true when repeat admissions were excluded.

Our investigation of non-psychiatric hospitalisations is, we believe, a novel for SMI and was made possible because of data linkages negotiated and set up over a long period with CRIS at the Maudsley NIHR Biomedical Research Centre. Other studies of hospitalisations have tended to focus either on mental healthcare specifically or all hospitalisations more generally (Jacobs *et al*. 2015; Kisely *et al*. 2015). In our study, considering individual diagnoses, clearly, a number of these hospitalisations outside mental health services attracted a primary diagnosis of a mental or behavioural condition, with alcohol-related disorders accounting for around a third of instances and the most common specific (three-digit) diagnosis. High SARs were observed for both affective and non-affective disorders, although those for the former were significantly higher (with non-overlapping confidence intervals). The role of harmful drinking as a cause of hospitalisations requires further more detailed evaluation in SMI but is likely to encompass a range of factors including admissions for intoxication or alcohol poisoning, inpatient detoxification episodes and potentially other health consequences assigned as alcohol-related in the recording of the primary diagnosis. Other hospitalisations due to direct impacts of underlying mental disorders are likely to include injuries, poisonings and other external causes arising secondary to self-harm and/or violence from others. Relationships between mental disorder and violence have been discussed extensively. A recent mental health policy paper that reviewed relevant literature concluded that SMI in itself does not cause an individual to be violent and instead it is the socio-demographic and socio-economic factors that lead to violence; the review also concluded that individuals with SMI are more likely to be victims of violence than perpetrators (Stuart, 2003). Substance misuse is clearly an important risk factor for perpetration and experience of violent behaviour both with and without co-occurring SMI (Stuart, 2003) and strategies to reduce this comorbidity may have potential benefits on a wide range of outcomes.

It is also interesting that hospitalisations assigned miscellaneous diagnostic codes occurred more often than expected in the SMI cohort, particularly those with non-affective disorders. These might reflect clinically unexplained symptoms or admissions precipitated by breakdowns in care arrangements and/or social support rather than secondary to a defined physical disorder. A recent project found that a small number of specialist mental health hospitals frequently used the ICD-10 code R69.x (‘unknown diagnosis’) as the primary diagnosis for people with a previous SMI diagnosis; however, we did not identify any literature to suggest that this was common practice within non-psychiatric settings (White *et al*. 2014). Clinically unexplained symptoms underlying non-specific codes are possibly supported by the higher frequencies of throat/chest pain and abdominal/pelvic pain. However, it should be borne in mind that pain in the throat and chest might have been caused by cardiac disease and it is well established that people with SMI have excess cardiovascular mortality (Leucht *et al.* 2007), suggesting a potential need for higher scrutiny of these symptoms, which might be complex to interpret when comorbid with mental disorders. Oesophageal reflux symptoms have been found to occur more frequently among people with a range of mental disorders, not just SMI, potentially indicating a reduced threshold for or distorted perception of symptoms (Avidan *et al*. 2001), although it is also important to bear in mind reflux associated with use of psychotropic drugs with sedative and/or anticholinergic actions, as well as an indirect effects of adverse lifestyle factors such as alcohol and substance misuse (Avidan *et al*. 2001).

Chronic renal failure predominated as a diagnosis for all admissions over the 2-year follow-up period, but this was accounted for by many admissions in a small number of patients, most likely representing inpatient dialysis episodes and/or admissions for complications of renal disease. The fact that people with SMI had both higher than expected total numbers of admissions with chronic renal failure and were more likely to have at least one admission with this condition, indicates a higher prevalence and severity of renal disease in this population. Other studies have also shown that in people with the chronic renal disease, co-occurring SMI can lead to an increased risk of experiencing another hospitalisation, particularly emergency admissions (McPherson *et al.* 2014). This may be accounted for by a higher burden of underlying risk factors such as hypertension and diabetes, although the role of medications such as lithium needs further evaluation, as does the possibility of reverse causation through long-term renal problems leading to the development of psychotic illness (Fanton *et al.* 2011; Tzeng *et al.* 2015). Of note, SARs for chronic renal failure were raised for both non-affective and affective disorders, but were significantly stronger in the former, which might reflect higher morbidity from underlying causes and/or difficulties negotiating the complex care required.

While the findings above are in line with the published literature, the association we found with higher than expected sickle cell related hospitalisations is possibly the first of its kind. Sickle cell disease mainly affects people of African, Caribbean, Middle Eastern, Eastern Mediterranean and Asian origin and the high SARs in our cohort are concerning, given the ethnic profile of the comparator local general population (around 25% black and black British residents). We could not identify any supporting literature beyond a handful of case reviews of psychosis in patients with sickle cell, thus indicating the need for further research. Of note, there was no substantial difference observed in SARs between affective and non-affective disorders. Another novel finding is that cataracts were less common as a discharge diagnosis in SMI compared with the general population which might reflect a lack of access to health services since we are not aware of any mental disorder profiles which would confer protection. However, coding issues need to be considered further, as we identified a high number of ‘other cataracts’ (ICD-10 code: H26). Studies elsewhere have identified that surgery for cataracts is more likely to happen as an inpatient for individuals with SMI than a day case where discharge is on the same day; therefore, we would have expected to see more and not fewer cataracts (Dorning *et al*. 2015).

There were also collections of discharge diagnoses potentially indicative of social deprivation and lack of health service access. These included dental caries, chest infections, and COPD, with smoking also a potential underlying factor. Dental caries also known as tooth decay is one of the two most common diseases that affect oral health, and people with SMI have been found to have over three times the odds of edentulousness than the general population (Kisely *et al*. 2011). The reasons for higher susceptibility to oral disease include smoking, amotivation syndromes, worse oral hygiene, generalised anxiety or a specific fear of dental examinations and procedures, costs of dental care, difficulty in accessing healthcare facilities, and side effects of psychotropic drugs such as xerostomia (Kisely *et al*. 2011). However, one study revealed that poor oral hygiene and reduced access to dental care were most important in determining the sub-optimal oral health of individuals with SMI (Matevosyan, 2010). Higher hospitalisations with pneumonia, organism unspecified, may also arise from self-neglect and impaired ability to recognise worsening of symptoms. In this respect, a lack of prompt detection and appropriate prevention of pneumonia have previously been found to increase the risk of poor outcomes including hospitalisation in schizophrenia (Yi-Hua *et al*. 2011). Poor treatment outcomes could also reflect misinterpretations of patients’ complaints as psychosomatic, resulting in delayed recognition (Schoepf *et al*. 2014). Self-neglect and its impact is an important consideration; however, we acknowledge here that the term ‘self-neglect’ is loosely applied, and definitions have not yet been established or validated, despite some attempts to clarify its contribution to vulnerability (Lamkin *et al*. 2016). Non-specific coding may also be an indication that individuals with SMI may need more advocacy in order to gain equitable access to appropriate care and support, and this might involve assistance in articulating what would otherwise be classified as unexplained symptoms.

This study had several strengths, including the large SMI cohort and its naturalistic data on people receiving mental and physical healthcare in an urban/ suburban neighbourhood. The outcome investigated was derived from a data source used in all national hospitals and was available for the whole of England. It should, therefore, have covered the vast majority of hospitalisations experienced both by the SMI cohort and the catchment population. The data linkage also provided the opportunity to separate out admissions only to non-psychiatric hospitals which, as mentioned, have received surprisingly little previous investigation. However, there are also limitations which need to be borne in mind. The study's focus was on people who had received an SMI diagnosis (schizophrenia, schizoaffective disorder or bipolar disorder) whether or not they had other mental health diagnoses as well. We did not seek to investigate other conditions, such as substance use disorders (F10–F19) and depressive disorders (F32–F33), although it is important to emphasise that they were not specifically excluded – i.e. comorbidity was allowable in the sample. The study was not able to distinguish balanced positive/negative effects at different points on the causal pathway; for example, breast cancer could conceivably be more common in SMI but inpatient healthcare less often sought/received. Furthermore, the study was restricted to assessing hospitalisations and did not attempt to investigate primary care outcomes or use of outpatient services. The study only investigated admissions to acute hospitals and there may, of course, have been individuals seen in mental health units for some physical illnesses. In addition, although analyses were carried out which excluded repeat presentations with the same diagnoses, we were not able to exclude repeat presentations with different diagnoses assigned. Finally, although the cohort is likely to be representative of people with SMI living in urban and suburban settings, there may be some unique features; for example, the prominence of sickle cell disease as a reason for hospitalisation may simply reflect the different ethnic compositions between the catchment general population and study cohort with SMI.

Non-psychiatric hospitalisation is an important outcome for a number of reasons. First, and most importantly, preventable admissions are likely to be occurring which are not in the best interest of the patient – these include readmissions which might be prevented through interventions delivered at the time of initial presentation. Trials identifying interventions found that using a complex and supportive strategy to assess and address contextual issues and limitations in patient capacity were most effective at reducing early hospital readmissions (Leppin *et al*. 2015). More specifically, where health professionals contacted the patient frequently and used home visits was beneficial and there is value in interventions that support patients’ capacity for self-care in their transition from hospital to home (Leppin *et al*. 2015). Second, preventable hospitalisations cost money that could be utilised differently for benefit of patients and public. Economic evaluations assessing quality interventions designed to reduce readmissions measuring the risk difference is readmission rates and incremental net cost found interventions that engaged patients and caregivers were associated with greater net savings (Nuckols *et al*. 2017). The same review found that diverse interventions can be effective at reducing readmissions, but cost savings do not consistently occur- it is the interventions that engage patients and family members that may be associated with larger net savings (Nuckols *et al*. 2017).

Our study highlights that there is a range of disease groups (e.g. digestive and respiratory conditions), specific disorders (e.g. sickle cell disease and chronic renal failure) and associated scenarios (e.g. self-neglect and alcohol abuse) that are important for policy development. Future research could helpfully focus on identifying and evaluating appropriate models for delivering more effective care, particularly for the conditions where SARs were higher than expected. However, investigation is also warranted where admissions were lower than that of the general population, to ensure that unmet needs are not being missed, given the recognized vulnerability of people living with SMI.

## Supplementary material

For supplementary material accompanying this paper visit http://dx.doi.org/10.1017/S0033291718000284. click here to view supplementary material
