# Supplementary material for: Acute general hospital admissions in people with serious mental illness
Source: Psychol Med. 2018 Feb 28;48(16):2676–83. doi: 10.1017/S0033291718000284 (PMC6236443; doi:10.1017/S0033291718000284)
Supplement: Supplementary file 1 [file S0033291718000284sup001.doc]

**Supplementary Table 1. Age- and gender-standardised admission ratios (SARs) during 2009-10 in people with affective and non-affective disorders (N of all admissions = 8,622), compared to the source population in the same regions**

| Primary cause of admission (ICD-10 codes) | SAR (95% CI, number of admissions) | |
| --- | --- | --- |
|  | Non-affective | Affective |
| Infectious diseases (A00-B99) | 2.43 (1.85- 3.12, 60) | 1.35 (0.80- 2.14, 18) |
| Neoplasms (C00-D48) | 2.36 (2.13- 2.61, 378) | 2.57 (2.26- 2.91, 254) |
| Blood disorders (D50-D89) | 4.77 (4.05- 5.59, 154) | 4.04 (3.21- 5.01, 82) |
| Endocrine and metabolic diseases (E00-E90) | 5.13 (4.41- 5.93, 181) | 4.69 (3.80- 5.73, 96) |
| Mental and behavioural disorders (F00-F99) | 34.67 (30.06- 39.80, 202) | 35.69 (28.94- 43.54, 97) |
| Nervous system (G00-G99) | 2.62 (2.15- 3.15, 111) | 2.62 (2.02- 3.34, 65) |
| Ear conditions (H60-H95) | 0.56 (0.15- 1.43, 4) | 1.47 (0.54- 3.20, 6) |
| Eye conditions (H00-H59) | 0.98 (0.80- 1.17, 111) | 0.77 (0.57- 1.01, 51) |
| Circulatory system (I00-I99) | 1.76 (1.57- 1.96, 315) | 1.35 (1.13- 1.61, 129) |
| Respiratory system (J00-J99) | 3.64 (3.26- 4.06, 328) | 3.49 (3.00- 4.03, 183) |
| Digestive system (K00-K93) | 2.00 (1.85- 2.16, 633) | 1.94 (1.74- 2.16, 341) |
| Skin conditions (L00-L99) | 1.99 (1.64- 2.40, 110) | 1.59 (1.18- 2.10, 49) |
| Musculoskeletal system (M00-M99) | 0.90 (0.76- 1.05, 150) | 1.39 (1.16- 1.65, 130) |
| Genitourinary system: urinary conditions (N00-N39) | 15.74 (14.90- 16.61, 1,329) | 11.81 (10.85- 12.84, 557) |
| Genitourinary system: pelvis, genitals and breasts (N40-N99) | 1.34 (1.09- 1.63, 101) | 1.06 (0.81- 1.35, 64) |
| Pregnancy related (O00-O99) | 1.48 (1.23- 1.75, 129) | 1.37 (1.14- 1.63, 127) |
| Congenital abnormalities (Q00-Q99) | 0.40 (0.05- 1.43, 2) | 1.41 (0.38- 3.62, 4) |
| Symptoms, signs and findings, not elsewhere classified (R00-R99) | 2.50 (2.30- 2.70, 608) | 2.06 (1.83- 2.31, 290) |
| Injury (S00-T14) | 2.82 (2.46- 3.20, 231) | 2.85 (2.37- 3.41, 121) |
| Poisoning and other external causes (T15-T98) | 6.21 (5.42- 7.07, 227) | 6.77 (5.70- 7.98, 141) |
| Factors influencing health status and contact with health services (Z00-Z99) | 2.36 (2.10- 2.64, 297) | 1.90 (1.61- 2.22, 156) |

**Supplementary Table 2. Age- and gender-standardised admission ratios (SARs) during 2009-10 in people with affective and non-affective disorders (number of all admissions = 8,622), compared to the source population in the same regions, excluding repeat hospitalisations**

| Primary cause of admission (ICD-10 codes) | SAR (95% CI, number of admissions) | |
| --- | --- | --- |
|  | Non-affective | Affective |
| Infectious diseases (A00-B99) | 1.92 (1.41- 2.55, 47) | 1.13 (0.63- 1.86, 15) |
| Neoplasms (C00-D48) | 0.76 (0.63- 0.91, 122) | 0.63 (0.48- 0.80, 62) |
| Blood disorders (D50-D89) | 1.61 (1.20- 2.11, 52) | 1.13 (0.72- 1.70, 23) |
| Endocrine and metabolic diseases (E00-E90) | 3.68 (3.08- 4.37, 130) | 2.54 (1.90- 3.33, 52) |
| Mental and behavioural disorders (F00-F99) | 25.40 (21.48- 29.84, 148) | 25.39 (19.75- 32.13, 69) |
| Nervous system (G00-G99) | 1.70 (1.33- 2.14, 72) | 2.05 (1.53- 2.70, 51) |
| Ear conditions (H60-H95) | 0.60 (0.15- 1.43, 4) | 1.22 (0.40- 2.86, 5) |
| Eye conditions (H00-H59) | 0.67 (0.53- 0.84, 76) | 0.54 (0.38- 0.75, 36) |
| Circulatory system (I00-I99) | 1.12 (0.97- 1.29, 201) | 0.92 (0.81- 1.14, 88) |
| Respiratory system (J00-J99) | 2.59 (2.27- 2.94, 233) | 2.19 (1.81- 2.63, 115) |
| Digestive system (K00-K93) | 1.38 (1.25- 1.52, 437) | 1.24 (1.08- 1.42, 219) |
| Skin conditions (L00-L99) | 1.59 (1.28- 1.96, 88) | 1.40 (1.01- 1.88, 43) |
| Musculoskeletal system (M00-M99) | 0.70 (0.58- 0.84, 117) | 0.99 (0.80- 1.21, 93) |
| Genitourinary system: urinary conditions (N00-N39) | 1.88 (1.60- 2.20, 159) | 1.46 (1.14- 1.85, 69) |
| Genitourinary system: pelvis, genitals and breasts (N40-N99) | 0.94 (0.73- 1.19, 71) | 0.83 (0.61- 1.09, 50) |
| Pregnancy related (O00-O99) | 0.82 (0.64- 1.04, 72) | 0.72 (0.56- 0.92, 67) |
| Congenital abnormalities (Q00-Q99) | 0.40 (0.05- 1.43, 2) | 1.41 (0.39- 3.62, 4) |
| Symptoms, signs and findings, not elsewhere classified (R00-R99) | 1.86 (1.69- 2.03, 452) | 1.38 (1.20- 1.60, 195) |
| Injury (S00-T14) | 2.32 (2.00- 2.67, 190) | 2.50 (2.05- 3.03, 106) |
| Poisoning and other external causes (T15-T98) | 3.74 (3.14- 4.43, 137) | 4.89 (3.99- 5.94, 102) |
| Factors influencing health status and contact with health services (Z00-Z99) | 1.96 (1.73- 2.22, 247) | 1.45 (1.20- 1.73, 119) |

**Supplementary Table 3. Age- and gender-standardised admission ratios (SARs) for three-character ICD code diagnoses contributing to at least 1% of all hospitalisations during 2009-10 among people with affective and non-affective disorders, compared to the source population in the same regions**

| ICD-10 code | Denoting clinical syndrome | SAR (95% CI, number of admissions) | |
| --- | --- | --- | --- |
|  |  | Non-affective | Affective |
| N18 | Chronic renal failure | 1.72 (1.62- 1.83, 1120) | 1.38 (1.26- 1.51, 472) |
| Z04 | Examination and observation for other reasons | 7.43 (6.40- 8.58, 186) | 5.34 (4.22- 6.66, 78) |
| K02 | Dental caries | 3.02 (2.57- 3.52, 163) | 1.78 (1.35- 2.29, 59) |
| N39 | Other disorders of urinary system | 3.04 (2.52- 3.63, 120) | 2.05 (1.54- 2.68, 53) |
| R07 | Pain in throat and chest | 1.72 (1.41- 2.08, 107) | 1.29 (0.93- 1.74, 42) |
| J18 | Pneumonia, organism unspecified | 3.34 (2.67- 4.09, 89) | 3.33 (2.51- 4.34, 55) |
| C50 | Malignant neoplasm of breast | 0.68 (0.50- 0.90, 50) | 0.77 (0.57- 1.01, 50) |
| R10 | Abdominal and pelvic pain | 1.67 (1.31- 2.10, 74) | 1.39 (1.00- 1.88, 41) |
| D57 | Sickle-cell disorders | 8.68 (6.78- 10.95, 71) | 9.69 (7.02- 13.06, 43) |
| E11 | Type 2 diabetes mellitus | 5.10 (4.03- 6.37, 77) | 3.19 (2.04- 4.74, 24) |
| F10 | Mental and behavioural disorders due to use of alcohol | 12.33 (9.15- 16.25, 50) | 27.86 (19.99- 37.80, 41) |
| J44 | Other chronic obstructive pulmonary disease | 2.11 (1.62- 2.70, 63) | 1.41 (0.91- 2.08, 25) |
| H26 | Other cataract | 0.52 (0.40- 0.68, 58) | 0.44 (0.29- 0.63, 29) |
